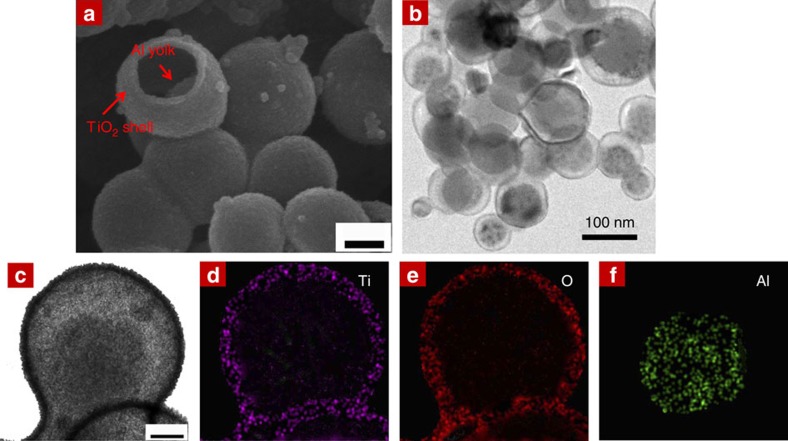# Corrigendum: High-rate aluminium yolk-shell nanoparticle anode for Li-ion battery with long cycle life and ultrahigh capacity

**DOI:** 10.1038/ncomms16174

**Published:** 2017-11-29

**Authors:** Sa Li, Junjie Niu, Yu Cheng Zhao, Kang Pyo So, Chao Wang, Chang An Wang, Ju Li

Nature Communications 6: Article number: 7872; DOI: 10.1038/ncomms8872 (2015); Published 08 05 2015; Updated 11 29 2017

This Article contains an error in which the scanning electron microscope image shown in Fig. 2b was included incorrectly. The original Fig. 2b showed yolk-shell microstructures, as previously published in Li, S. and Wang, C.-A. Design and synthesis of hierarchically porous MnO_2_/carbon hybrids for high performance electrochemical capacitors. *Journal of Colloid and Interface Science* **438**, 61–67 (2015). (©2014, with permission from Elsevier). We and our colleague Dr Yuming Chen have repeated the high-rate battery cycling tests of the material, which is supplied in the Supplementary Information associated with this correction, and this error does not have consequence on the science, data or conclusions of the Article, but we apologize for any confusion caused. The correct version of Fig. 2, which shows the inner aluminum yolk encapsulated by 2–4 nm-thick TiO_2_ shell in the revised panel **b**, appears below as Fig. 1.

## Figures and Tables

**Figure 1:**